# Real-time monitoring of the reaction of KRAS G12C mutant specific covalent inhibitor by in vitro and in-cell NMR spectroscopy

**DOI:** 10.1038/s41598-023-46623-w

**Published:** 2023-11-07

**Authors:** Qingci Zhao, Ryoka Haga, Satoko Tamura, Ichio Shimada, Noritaka Nishida

**Affiliations:** 1 Graduate School of Pharmaceutical Sciences, Chiba University, 1-8-1 Inohana, Chuo-ku, Chiba, 260-8675 Japan; 2 RIKEN Center for Biosystems Dynamics Research, 1-7-22 Suehiro-cho, Tsurumi-ku, Yokohama, Kanagawa 230-0045 Japan; 3 Graduate School of Integrated Sciences for Life, Hiroshima University, Higashi-Hiroshima, 739-8528 Japan

**Keywords:** Solution-state NMR, Chemical modification

## Abstract

KRAS mutations are major drivers of various cancers. Recently, allele-specific inhibitors of the KRAS G12C mutant were developed that covalently modify the thiol of Cys12, thereby trapping KRAS in an inactive GDP-bound state. To study the mechanism of action of the covalent inhibitors in both in vitro and intracellular environments, we used real-time NMR to simultaneously observe GTP hydrolysis and inhibitor binding. In vitro NMR experiments showed that the rate constant of ARS-853 modification is identical to that of GTP hydrolysis, indicating that GTP hydrolysis is the rate-limiting step for ARS-853 modification. In-cell NMR analysis revealed that the ARS-853 reaction proceeds significantly faster than that in vitro, reflecting acceleration of GTP hydrolysis by endogenous GTPase proteins. This study demonstrated that the KRAS covalent inhibitor is as effective in the cell as in vitro and that in-cell NMR is a valuable validation tool for assessing the pharmacological properties of the drug in the intracellular context.

## Introduction

RAS is a representative member of the small GTPase family that mediates signal transduction downstream of receptor tyrosine kinases to regulate cellular functions, such as cell growth, motility, and survival^1^. RAS exchanges between a GDP-bound inactive state and a GTP-bound active state. The conversion of the GDP-bound to GTP-bound state induces a conformational change in RAS that enables interaction with downstream effector molecules, such as Raf, RalGDS, and PI3K^2, 3^. Intrinsic or GTPase activating protein (GAP)-mediated GTP hydrolysis inactivates RAS in the GDP-bound state^4^. Hotspot mutations at Gly12, Gly13, and Gln61, which lead to the constitutive activation of RAS, are frequently found in various cancer types, making RAS a prominent target for cancer therapeutics^1^.

Despite decades of intensive efforts, RAS-targeting therapies have not reached clinical application until recently. Many competitive inhibitors for various kinases have been developed, targeting the ATP-binding site or surrounding pocket^5^. By contrast, RAS has an extremely high affinity for GTP (Kd ~ 20 pM) and a high intracellular concentration of free GTP (submillimolar range), making competitive inhibitors targeting the GTP-binding pocket of RAS impractical. In addition, no clear pockets exist on the effector-binding surface of active RAS that can accommodate compounds. Therefore, RAS has been an undruggable target. Several groups have developed compounds that directly bind RAS and inhibit interactions with downstream effector molecules using fragment-based drug design and in silico approaches^6–10^. However, these compounds could not efficiently inhibit oncogenic RAS mutants in cells and in vivo.

A breakthrough in RAS-targeting drug discovery was achieved for the KRAS G12C mutant, which accounts for 12% of all cancer-associated KRAS mutations^11^. Shokat and co-workers developed several compounds covalently attached to the thiol group of C12 using an acrylamide warhead. The remaining moieties of the inhibitors is accommodated within the pocket under the Switch II region (termed S-II pocket), which is formed upon inhibitor binding^12^. The biochemical study indicated that those covalent inhibitors bind KRAS G12C in the GDP-bound state, strongly inhibiting GDP–GTP exchange even in the presence of guanine nucleotide exchange factors (GEFs)^13^. In vitro, ARS-853 could interact with KRAS G12C in the GDP-bound form with high potency but low binding affinity (K_i_ ~ 200 µM)^14^. Such characteristic potency of ARS-853 is explained by the stabilization of the transition state through formation of electrostatic interactions between the protonated K16 of KRAS and the acrylamide warhead. Subsequent efforts to enhance efficacy and pharmacological properties have led to the development of a range of related compounds, which have exhibited significant effects in vivo and in a xenograft mouse model^15^, and clinical trials are being conducted by multiple independent companies^16^. Two compounds, AMG 510 (sotorasib) and MRTX-849 (adagrasib), have received FDA approval for treating NSCLC^17^. This is a significant milestone, because these drugs are the first to target mutated KRAS, offering hope to patients.

The intracellular reactivity of covalent inhibitors of KRAS is affected by various cellular factors. Many KRAS mutants, including G12C, exhibit impaired intrinsic and GAP-mediated GTP hydrolysis activity^18^, which is unfavorable for covalent inhibitors that react with the GDP-bound form. It was recently reported that the endogenous proteins markedly enhances the GTP hydrolysis of KRAS mutants to increase the intracellular reactivity of the covalent inhibitors^19^. However, intracellular molecular crowding could negatively affect protein–drug interactions due to unwanted nonspecific interactions with endogenous molecules^20^. In addition, recent study demonstrated that the reactivity of the covalent inhibitor was affected by solution pH, owing to the suppressed pKa value of the C12 thiol^21^. Therefore, to evaluate the intracellular activity of the covalent inhibitors of KRAS, it is necessary to monitor the progress of GTP hydrolysis of KRAS and formation of covalent bonds under the macromolecular crowding environments, where proteins and metabolites are present at high concentrations.

Solution NMR allows us to observe chemical and enzymatic reactions in real-time under physiological conditions^22^. Previously, the GTP hydrolysis rate (*k*_hy_) and GDP–GTP exchange rate (*k*_ex_) of small GTPases, including RAS, were characterized by real-time observations of NMR signals derived from the GTP- and GDP-bound forms^23^. Real-time NMR can be performed without fluorescent nucleotide analogs^24^, ^32^P radio isotope-labeled nucleotides, or effector-based interactions. Our group exploited the in-cell NMR method to monitor the level of GTP-bound level of HRAS in HeLa S3 cells and directly quantified *k*_hy_ and *k*_ex_ for HRAS and its oncogenic mutants^25^. Therefore, NMR enables simultaneous observation of the progress of GTP hydrolysis and the reaction of the covalent inhibitor both in vitro and intracellularly. In this study, we investigated the kinetics of the reaction of ARS-853 and GTP hydrolysis of KRAS G12C using in vitro and in-cell NMR.

## Results

### Characterization of the G12C mutant and ARS-853 modification

**Figure 1:**
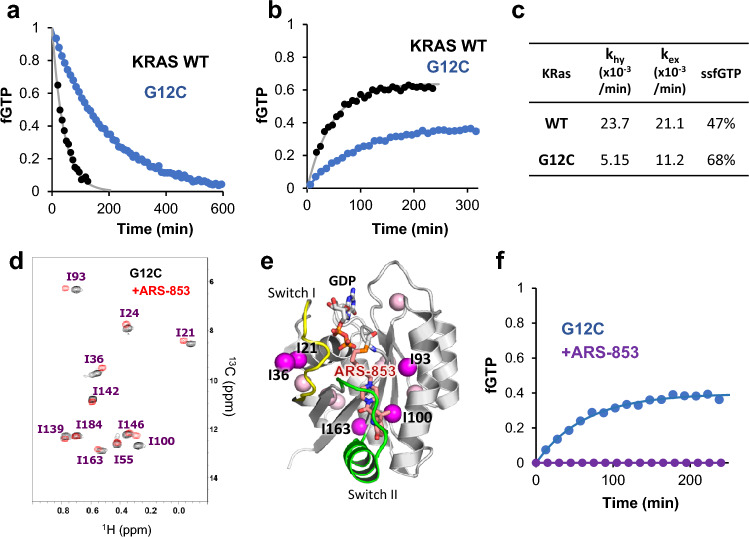
Characterization of the KRAS G12C mutant and its conjugation with ARS-853. (**a**,**b**) Measurements of GTP hydrolysis (**a**) and GDP–GTP exchange (**b**) rates of KRAS WT and G12C mutant. Time-dependent changes of fGTP for WT (gray circles) and G12C (blue circles) were measured based on NMR signal intensities of I21 signals and subjected to single exponential curve fitting (solid lines). (**c**) Summary of in vitro parameters of KRAS WT and G12C. The theoretical fraction of the GTP-bound state at steady state (ssfGTP) was calculated using experimental *k*_hy_ and *k*_ex_ values. (**d**) Overlaid ^1^H–^13^C HSQC spectra of KRAS G12C with (red) and without (black) ARS-853 modification. (**e**) Mapping of chemical shift difference for Ile residues (spheres) caused by ARS-853 modification. The Ile δ1 methyl groups showing significant chemical shift difference (δΔ = δH_2_ + (δC/5)^2^)^1/2^ is more than 0.002 ppm) are in magenta. (**f**) The GDP–GTP exchange profiles of G12C with (purple) and without (blue) ARS-853 modification. GDP-loaded KRAS G12C was incubated with 2 equimolar of GTPγS.

We evaluated *k*_hy_ and *k*_ex_ of KRAS wild-type (WT) and G12C mutant. As described in our previous study, the δ1 methyl signal of I21 signals in the ^1^H–^13^C hetero-nuclear single quantum coherence (HSQC) spectrum can be used to measure the fraction of GTP- and GDP-bound states based on their signal intensity^25^. We measured ^1^H–^13^C HSQC spectra of GTP-loaded KRAS (WT and G12C) continuously to estimate *k*_hy_ by single exponential curve fitting of the time-dependent change of the fraction of GTP-bound state (fGTP) (Fig. 1a). The *k*_hy_ of the G12C mutant was 5.15 × 10^−3^ min^−1^, which is 4.6 times lower than that of KRAS WT. We estimated *k*_ex_ by measuring a series of ^1^H–^13^C HSQC spectra of GDP-loaded KRAS in the presence of 2 molar excess of nonhydrolyzable GTP analog (GTPγS) (Fig. 1b). The estimated *k*_ex_ of G12C was 1.12 × 10^−2^ min^−1^, 1.9 times slower than KRAS WT. Based on *k*_hy_ and *k*_ex_, fGTP in the steady state (ssfGTP) can be calculated as *k*_ex_/(*k*_hy_ + *k*_ex_). Due to the reduction of *k*_hy_ and decrease of *k*_ex_, the ssfGTP of G12C was elevated to 68% compared with KRAS WT (47%) (Fig. 1c). We estimated *k*_hy_ and *k*_ex_ using the GTP regeneration system, in which GDP released from RAS following GTP hydrolysis was regenerated to GTP by acetate kinase. Because the concentration of free GTP can be kept constant in the GTP regeneration system, *k*_hy_ and *k*_ex_ values can be simultaneously estimated by curve fitting of the time course of fGTP (Supplementary Fig. 1a). *k*_hy_ and *k*_ex_ values were almost identical with those obtained from individual experiments (Supplementary Fig. 1b).

Next, we analyzed the properties of KRAS G12C modified with ARS-853. Mass spectrometry analysis showed that treatment of GDP-loaded KRAS G12C with two equimolar ARS-853 for 2 h led to a remarkable increase in mass (Supplementary Fig. 2). Comparison of ^1^H–^13^C HSQC spectra of KRAS G12C with or without ARS-853 showed significant chemical shift changes for Ile signals proximal to switch II pocket (I93, I100) and those in switch I loop (I21 and I36), indicating modification of ARS-853 and the resulting conformational change (Fig. 1d,e). ARS-853-conjugated KRAS exhibited no exchange to GTP-bound form in the presence of 2 molar excess GTPγS (Fig. 1f). Thus, the ARS-853-conjugated KRAS G12C mutant was trapped in the GDP-bound form.

### Real-time analysis of the ARS853 modification in vitro

**Figure 2:**
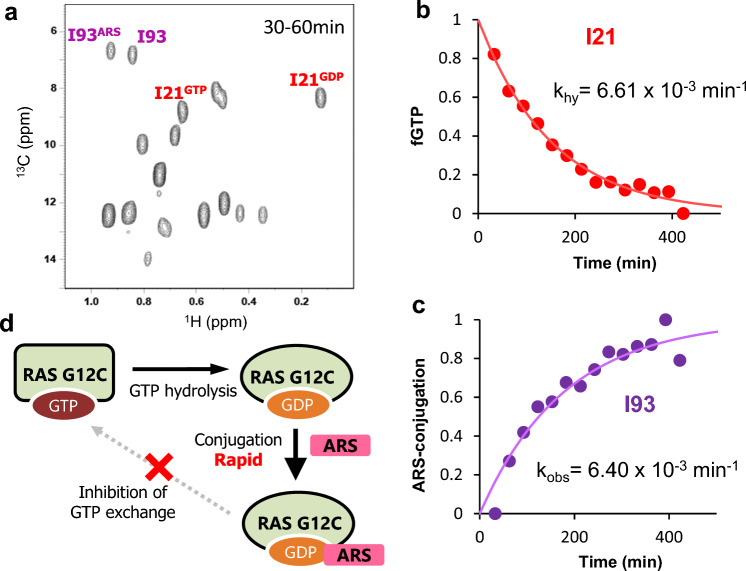
Real-time observation of ARS-853 reaction under in vitro conditions. (**a**) A representative ^1^H–^13^C HSQC spectrum of KRAS G12C acquired immediately after the addition of ARS-853. I21 signal (for GTP/GDP-bound ratio) and I93 signals (for the ARS-853 reaction) are labeled in red and purple, respectively. (**b**) Time course of ARS-853 modification, monitored based on I93 signal intensity. (**c**) Time course of GTP hydrolysis in the presence of ARS-853, monitored by I21 signal intensity. (**d**) Schematics of ARS-853 inhibition mechanism based on in vitro NMR experiments.

We monitored ARS-853 modification of KRAS G12C in real-time with in vitro NMR measurement. Based on the previous kinetic study showing that the binding affinity of ARS-853 is weak (Ki ~ 200 μM)^14^, we recorded the time-resolved ^1^H–^13^C HSQC spectra of GTP-loaded G12C in the presence of 8 molar excess of ARS-853 ([KRAS]:[ARS-853] = 50:400 μM) so that the measured rate constant (*k*_obs_) would be dominated by the reaction rate (*k*_inact_) (Fig. 2a). The progress of covalent bond formation was estimated by the intensity of I93 signals, which showed distinctive chemical shift changes upon ARS-853 modification (Fig. 2b). The rate constant of ARS-853 modification was 6.40 × 10^−3^ min^−1^. The progress of GTP hydrolysis of KRAS G12C in the presence of ARS-853, monitored by I21 signals, was 6.61 × 10^−3^ min^−1^ (Fig. 2c). Thus, *k*_hy_ was almost identical to the rate of ARS-853 modification, indicating that the ARS-853 reaction occurs immediately after GTP hydrolysis. In addition, there is no significant difference in *k*_hy_ values in the presence or absence of ARS-853. Therefore, it was verified that the presence of ARS-853 does not affect the GTP hydrolysis of RAS G12C and GTP hydrolysis is the rate-limiting step for ARS-853 to conjugate the G12C thiol group (Fig. 2d).

### Real-time analysis of ARS-853 modification in the intracellular environment

**Figure 3:**
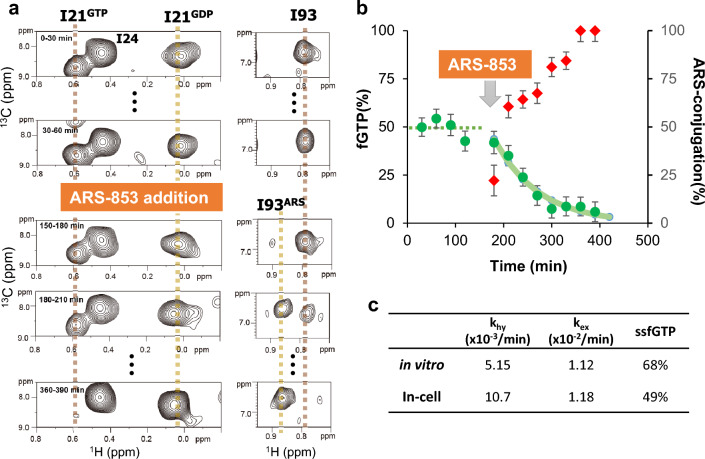
In-cell NMR analysis of the ARS-853 modification within the cells. (**a**) Time-resolved observation of I21 (left panels) and I93 (right panels) signals before and after ARS-853 addition. (**b**) Time-dependent changes of fGTP before and after ARS-853 addition (green circles) and the rate of ARS-853 modification (red diamonds). Average intracellular fGTP in the absence of ARS-853 is indicated by a dotted line. Curve fitting for *k*_hy_ in the presence of ARS-853 is indicated by a solid green line. Error bars are estimated based on the signal-to-noise ratio. (**b**) Summary of KRAS G12C parameters (*k*_hy_, *k*_ex_, and ssfGTP) measured in vitro and in-cell.

To monitor ARS-853 modification intracellularly, we performed in-cell NMR analysis of KRAS G12C. To observe the NMR signal under intracellular conditions, stable isotope-labeled KRAS G12C was introduced into HeLa S3 cells by reversible membrane permeabilization using streptolysin O (SLO) (Supplementary Fig. 3a)^26^. The cells were encapsulated within the Mebiol gel thread inside the 5-mm NMR tube and continuously perfused with fresh culture medium using the bioreactor system during NMR measurements^27^. To monitor intracellular fGTP of G12C in the absence of ARS-853, ^1^H–^13^C SOFAST-HMQC^28^ (band-selective optimized flip angle short transient hetero-nuclear multiple quantum coherence) spectra were measured every 30 min under perfusion of medium without ARS-853 (Fig. 3a, Supplementary Figs. 3b and 4a). Intracellular fGTP of G12C was already reached the steady state, with 49.5% from the average of four time points (Fig. 3b). Although the ssfGTP of intracellular G12C was lower than that measured in vitro (68%), this result demonstrated that the G12C mutation caused constitutive activation intracellularly.

To observe the ARS-853 reaction inside cells, we replaced the perfusion medium with a medium containing 100 μM ARS-853 and resumed NMR measurements (Fig. 3a and Supplementary Fig. 4b). We observed the time-dependent decay of the GTP-bound signal of I21 (Fig. 3b). The I93 signal also exhibited a chemical shift change in a time-dependent manner, reflecting the ARS-853 modification (Fig. 3b). The ARS-853 modification monitored by I93 signals was almost comparable with the GTP hydrolysis probed by I21 signals, except for the first 30 min time point, which would reflect the delayed transportation of the inhibitor across the plasma membrane. Considering that the ARS-853 modification inhibits GDP–GTP exchange, the temporal change of intracellular fGTP represents the progress of GTP hydrolysis in the cells. The rate constant of GTP hydrolysis in cells estimated from the I21 signal was 1.09 × 10^−2^ min^−1^, which is a 2.1-fold increase compared with the in vitro rate, suggesting that the modification of ARS-853 proceeds faster than in vitro due to the increase in the intracellular *k*_hy_ of RAS. Based on intracellular *k*_hy_ (1.09 × 10^−2^ min^−1^) and ssfGTP values of the G12C mutant (49.5%), intracellular *k*_ex_ was estimated to be 1.18 × 10^−2^ min^−1^ (Fig. 3c). This estimation closely matched with in vitro, indicating that the *k*_ex_ of KRAS G12C is not significantly affected by the intracellular environment.

## Discussion

In this study, we performed real-time monitoring of covalent inhibitor targeting of KRAS G12C using in vitro and in-cell NMR. Based on the NMR signals of I21 and I93, which exhibited chemical shift changes upon conversion of the GTP/GDP-bound state and covalent bond formation with the inhibitor, respectively, the progress of GTP hydrolysis and ARS-853 modification can be simultaneously monitored by acquiring time-resolved NMR spectra. In vitro analysis revealed that the reaction between ARS-853 and KRAS G12C occurred in concert with the progress of GTP hydrolysis, thereby precluding the conversion of the GTP-bound activated form. This result directly demonstrated the postulated mechanism based on the mutational study^13^. In-cell NMR experiments confirmed that ARS-853 converts the constitutively activated KRAS G12C into the inactive GDP-bound form with a higher rate constant than in vitro.

In the previous study, the reaction of the covalent inhibitors was monitored by an LC–MS/MS-based method, which required cell disruption and subsequent sample preparation to apply mass spectrometry^29^. By contrast, the in-cell NMR method can directly monitor reaction progress in the native intracellular environment within living cells. One caveat of the current in-cell NMR study is that it used the KRAS G-domain without the C-terminal hypervariable region; therefore, RAS was not localized to the plasma membrane. Notably, ssfGTP of KRAS G12C was ~ 50%, which is consistent with the result of the RAS-binding domain (RBD)-MS assay performed in the presence of erlotinib (EGFR inhibitor)^29^. Therefore, the result of the in-cell NMR experiments would reflect the activity status of KRAS in the basal state without external cellular stimuli. The previous NMR study of full-length KRAS immobilized on nanodiscs showed that the Switch II region of RAS is exposed to the solvent on the lipid bilayer^30^, suggesting that the inhibitor binding is not affected by membrane localization of RAS. On the other hand, in order to observe the effects of inhibitors in the presence of receptor stimulation, it is necessary to perform in-cell NMR analysis under conditions where KRAS is localized to the membrane through lipid modifications, as recently reported^31^.

Compared with the in vitro rate, the intracellular reaction rate of ARS-853 was elevated due to the increase of the *k*_hy_ value of G12C. Increase of intracellular *k*_hy_ was also observed for HRAS mutants in our previous in-cell NMR study^25^. Although these mutants are known to be insensitive to canonical GAPs, such as RASA1 and NF1^18^, we previously found that unidentified intracellular proteins with molecular weight 30–50 kDa could increase *k*_hy_^25^. Consistently, a study reported that the GTP hydrolysis of G12C is enhanced by RGS3 (a GAP that regulates heterotrimeric G-proteins), which possesses catalytic mechanisms distinct from canonical GAPs^19^. Although the expression level of RGS3 in HeLa cells has been reported to be low^32^, it is expected that other atypical GAPs contribute to the enhancement of GTP hydrolysis of G12C mutant.

One of the major problems in the early stages of drug development is that the drug candidates obtained by in vitro screening often fail at the cellular level due to low membrane permeability or poor intracellular binding selectivity. In-cell NMR experiments fill the gap between in vitro and cellular level assays, allowing direct assessment of drug efficacy under intracellular conditions, including the membrane permeabilization efficiency. Several in-cell NMR studies have demonstrated that protein–drug interactions can be evaluated quantitatively under intracellular conditions^33, 34^. We expect that in-cell NMR will be a widely applied tool for assessing the pharmacological properties of a drug at early stages of drug development.

## Methods

### Protein preparation

The cDNA encoding KRAS G-domain (residues 1–169) was cloned into the pET15b vector and transformed into *E. coli* BL21 (DE3) strain. Protein expression was induced by 0.4 mM IPTG for 16 h at 25 °C. Cells were harvested and lysed by sonication, and cell debris and insoluble fraction were removed by centrifugation. The supernatant was purified by nickel-NTA affinity chromatography, and further purified by Superdex 75 size exclusion chromatography (GE healthcare). Stable isotope-labeled [uniform (U)-[^2^H], Ileδ1-[^13^C^1^H_3_]] KRAS was prepared as described^35^. The KRAS G12C mutant was generated using the QuikChange method (Agilent technologies). To prepare GTP-loaded KRAS, GDP-bound KRAS in HEPES buffer (20 mM HEPES [pH 7.2], 150 mM NaCl, 5 mM MgCl_2_, and 1 mM TCEP) was incubated with 10 mM EDTA and 5 mM GTP (Sigma) for 10 min to strip GDP. Then 15 mM MgCl_2_ was added for GTP rebinding. Free excess GTP/GDP was removed by dialysis against HEPES buffer. A 10 mM stock solution of ARS-853 (SelleckChem) was prepared in DMSO.

### In vitro NMR measurements

In vitro NMR experiments were performed either by Bruker Avance 500 or Avance 600 NEO spectrometer equipped with a cryoprobe at 37 °C. To monitor GTP hydrolysis, a series of ^1^H–^13^C HSQC spectra were measured every 15 or 30 min for 200 μM KRAS loaded with GTP. For GDP–GTP exchange rate and ARS-853 modification, 50 μM GDP-loaded KRAS was mixed with 200 μM GTPγS or 400 μM ARS-853, respectively, and time-resolved ^1^H–^13^C HSQC spectra were recorded every 15 or 30 min. All spectra were processed by TopSpin 3.6 (Bruker), and the peak intensities of the GDP/GTP state were analyzed by Topspin 3.6 or UCSF SPARKY. The *k*_hy_ and *k*_ex_ values were estimated by non-linear curve fitting using Eqs. (1) and (2), respectively.

1 $${\left[Ra{s}_{GTP}\right]}_{t}={\left[Ra{s}_{GTP}\right]}_{0} exp(-{k}_{hy}*t)$$

2 $${\left[Ra{s}_{GTP}\right]}_{t}={\left[Ra{s}_{GDP}\right]}_{\infty } (1-exp\left(-{k}_{ex}*t\right))$$

where [RAS_GTP_]_t_ and [RAS_GDP_]_t_ denote the fraction of RAS in GTP- and GDP-bound forms, respectively, at time point *t*. The fraction of GTP-bound form at the steady state (ssfGTP) was calculated using Eq. (3).

3 $$ssfGTP=\frac{{k}_{ex}}{{k}_{hy}+{k}_{ex}}$$

### In-cell NMR measurements

HeLa S3 cells (JCRB#0713) were cultured in DMEM (supplemented with 10% FBS) under 5% CO_2_ atmosphere. In total, 1 × 10^8^ cells were treated with 20 ng/mL SLO at 4 °C for 10 min at cell density 2.0 × 10^6^ cells/mL and washed with HBSS buffer (30 mM HEPES–KOH [pH 7.2], 137 mM NaCl, 5.4 mM KCl, 0.25 mM Na_2_HPO_4_, 0.44 mM KH_2_PO_4_, 4.2 mM NaHCO_3_, 1% w/v d-glucose). The cells were gently mixed with 500 μL of 1.0 mM KRAS G12C in transport buffer (25 mM HEPES [pH 7.4], 115 mM KOAc, 5 mM MgCl_2_, 2 mM EDTA) at 37 °C for 30 min. The cells were resealed with HBSS containing 1 mM CaCl_2_, filtered with a 100-μm mesh to remove cell debris, washed two times with HBSS, mixed with 150 μL of 6% Mebiol gel at 4 °C, transferred to a 5-mm Shigemi tube as a coil-shaped thread using a Pasteur pipet, and incubated at 37 °C to form a gel.

All in-cell NMR experiments were performed at 37 °C using an AvanceIII HD 800 spectrometer equipped with a TCI probe (Bruker). ^1^H–^13^C SOFAST HMQC spectra were recorded with 30 min measurement time under perfusion with DMEM containing 20% D_2_O (without serum) at a flow rate of 2.75 mL/h. To measure the ARS-853 reaction in the cell, the inlet tube was detached from the NMR tube, purged with the medium containing 100 μM ARS-853, and then reattached. The cells were perfused with ARS-853 containing medium for at least 15 min at a flow rate of 2.75 mL/h before resuming subsequent NMR measurements.

### Supplementary Information

Supplementary Figures.

## Data Availability

The datasets generated during and/or analyzed during the current study available from the corresponding author on reasonable request.
