# Supplementary material for: Real-time monitoring of the reaction of KRAS G12C mutant specific covalent inhibitor by in vitro and in-cell NMR spectroscopy
Source: Sci Rep. 2023 Nov 7;13:19253. doi: 10.1038/s41598-023-46623-w (PMC10630485; doi:10.1038/s41598-023-46623-w)
Supplement: Supplementary file 1 — Supplementary Figures. [file 41598_2023_46623_MOESM1_ESM.docx]

Supplemental Information

**Real-time monitoring of the reaction of KRAS G12C mutant specific covalent inhibitor by in vitro and in-cell NMR spectroscopy**

Qingci Zhao^1^, Ryoka Haga^1^, Satoko Tamura^2^, Ichio Shimada^2,3^*, Noritaka Nishida^1^*

1 Graduate School of Pharmaceutical Sciences, Chiba University, 1-8-1 Inohana, Chuo-ku, Chiba 260-8675, Japan

2 RIKEN Center for Biosystems Dynamics Research, 1-7-22 Suehiro-cho, Tsurumi-ku, Yokohama, Kanagawa, 230-0045, Japan

3 Graduate School of Integrated Sciences for Life, Hiroshima University, Higashi-Hiroshima 739-8528, Japan


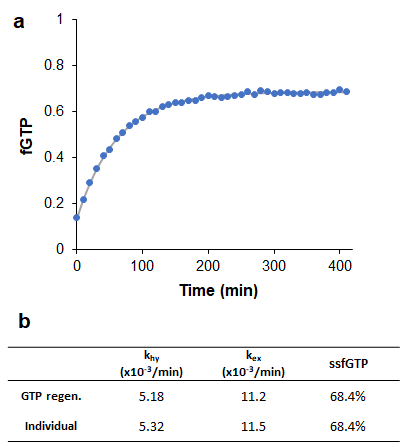


**Figure S1 Simultaneous estimation of k_hy_ and k_ex_ of KRAS G12C using GTP regeneration system.** (a) ^1^H-^13^C HSQC spectra were recorded every 10 min at 37°C using the sample containing 200 μM KRAS G12C in the GTP bound form in the presence of 100 mU/mL acetate kinase (AcK) and 10 mM acetyl phosphate (AcP) (Sigma Aldrich), 2-fold excess (400 μM) of GTP. The plot of fGTP was fitted with the following equation to estimate k_hy_ and k_ex_ simultaneously.

$${[fGTP]}_{t}=\frac{k_{ex}\left( 1-\exp\left( -\left( k_{hy}+k_{ex} \right)t \right) \right)+(k_{hy}+k_{ex})\left[ fGTP \right]_{0}\exp\left( -\left( k_{hy}+k_{ex} \right)t \right)}{k_{hy}+k_{ex}}$$

Where [fGTP]_t_ denotes the fGTP at the time t.

(b) Summary of the k_hy_, k_ex_, and steady state fGTP obtained from individual and GTP regeneration experiments.


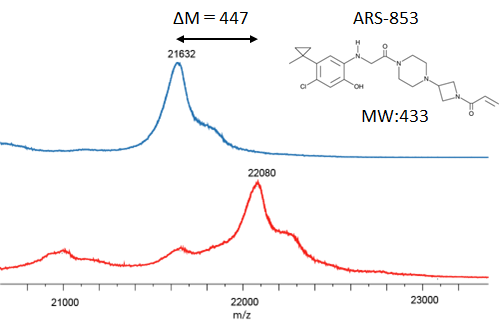


**Figure S2 Modification of ARS-853 confirmed by MALDI-TOFMS.**

100 μM KRAS G12C in the GDP-bound form was treated with 200 μM ARS-853 for 2 hours in 20 mM HEPES (pH 7.0), 100 mM NaCl, 5 mM MgCl_2_, and 1 mM TCEP. Samples were de-salted by ZipTip and mixed with 10 mg/mL sinapinic acid containing 0.1 % TFA, and subjected to MALDI-8020 mass spectrometry (Shimadzu). Compared to the untreated sample (top), an increase of mass corresponding to 1 unit of ARS-853 (MW 433) was observed after the ARS-853 treatment (bottom). Minor peaks are derived from partially degraded products.


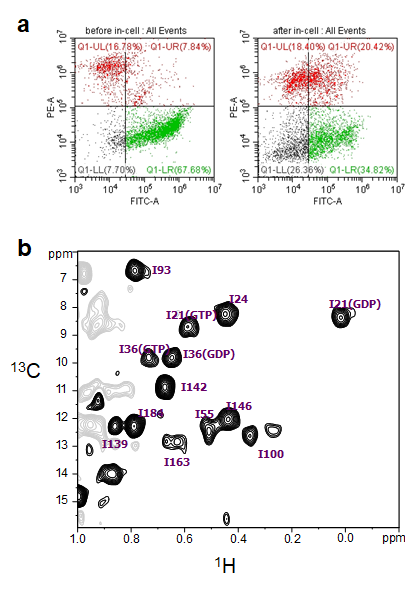


**Figure S3 In-cell NMR experiments of KRAS G12C using HeLa S3 cells**

**(a)** Representative FCM plots of RAS-introduced cells before (left) and after (right) the in-cell NMR measurement. FITC-labeled RAS were co-introduced with isotope-labeled RAS for estimating the population of KRAS-introduced cells (green plots). Cells were stained with propidium iodide (PI) to estimate the viability during the measurements (red plots). (b**)** ^1^H-^13^C SOFAST HMQC spectrum of the GTP-loaded KRAS G12C introduced cells at the first 30 min. The positive signals are shown black, while the aliased negative signals are colored gray. The signals with ^1^H chemical shift above 0.8 ppm are background signals derived from the medium or endogenous cellular proteins. All eleven Ile signals were observed, and their chemical shift patterns were similar to those observed *in vitro*, without overlapping background signals except for I139.


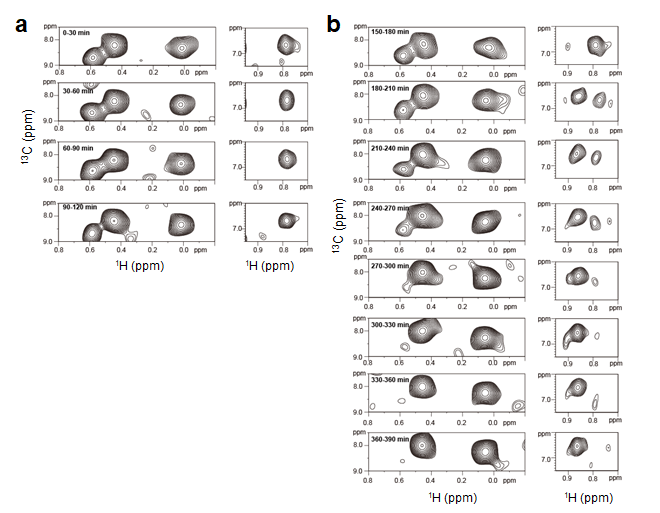


**Figure S4**

The full time-course of in-cell NMR signals of Ile21 and Ile93 in the absence (a) and presence (b) of ARS-853 in the circulating medium. The medium exchange was performed during the 120-150 min interval.
